# Effects of Skin Stimulation on Sensory-Motor Networks Excitability: Possible Implications for Physical Training in Amyotrophic Lateral Sclerosis

**DOI:** 10.3389/fneur.2022.868792

**Published:** 2022-05-25

**Authors:** Marco Ceccanti, Chiara Cambieri, Laura Libonati, Giorgio Tartaglia, Federica Moret, Matteo Garibaldi, Maurizio Inghilleri

**Affiliations:** ^1^Department of Human Neuroscience, Center for Rare Neuromuscular Diseases, Policlinico Umberto I, Sapienza University of Rome, Rome, Italy; ^2^Department of Neuroscience, Mental Health and Sensory Organs (NESMOS), Neuromuscular and Rare Disease Center, Sant'Andrea Hospital, Sapienza University of Rome, Rome, Italy

**Keywords:** paired associative stimulation, sensory-motor networks, amyotrophic lateral sclerosis, brain stimulation, cortical excitability

## Abstract

**Background:**

Many different trials were assessed for rehabilitation of patients with amyotrophic lateral sclerosis (ALS), with non-unique results. Beside the effects on muscle trophism, some of the encouraging results of physical training could be ascribed to the modulation of cortical excitability, which was found hyperexcited in ALS.

**Objective:**

The effects of tactile skin stimulation in the modulation of the sensory-motor integrative networks in healthy subjects were assayed through the paired associative stimulation (PAS) protocol.

**Methods:**

In total, 15 healthy subjects were enrolled. In the *standard PAS session*, the average amplitude of the motor evoked potential (MEP) after 10 stimuli of transcranial magnetic stimulation (TMS) was measured at the baseline and after the PAS protocol (0, 10, 20, 30, and 60 min). In the *skin stimulation session*, the average amplitude of the MEP was measured before and after 10 min of skin stimulation over the hand. Subsequently, each subject underwent the PAS stimulation and the measure of the average amplitude of the MEP (0, 10, 20, 30, and 60 min).

**Results:**

The tactile skin stimulation on healthy subjects increases the PAS-induced sensory-motor network hyperexcitability in healthy subjects.

**Conclusion:**

Skin stimulation should be avoided in the physiotherapeutic approaches for patients with ALS, given the possible hyperexciting effects on the already upmodulated sensory-motor networks. They can be taken into account for diseases characterized by downregulation of cortical and transcortical networks.

## Introduction

Amyotrophic lateral sclerosis (ALS) is a rare, adult-onset neurodegenerative disease characterized by a loss of motor neurons in the cerebral cortex, brainstem, and spinal cord, giving rise to upper and lower motor neuron signs. No effective therapy is available, even if some drugs demonstrated a mild effect in slowing down the disease progression (1, 2). Even more challenging is the etiopathology of ALS; genetic (3), autophagy (4, 5), glutamate-driven excitotoxicity in the motor cortex (6, 7), oxidative stress (8), mitochondrial dysfunction (9), muscle impairment (10), and many other mechanisms have been considered.

Many studies evidenced non-motor involvement in motor neuron diseases, such as gastrointestinal dysfunction (7), cognitive impairment (11), extrapyramidal signs (12–15), small fiber neuropathy (16), and laryngeal sensitivity (17). Moreover, sensory-motor networks are demonstrated to be impaired in superoxide dismutase 1 (SOD1) ALS mice, which exhibit specific delays in acquiring sensory-motor skills even during the first week after birth (18). All these data are designing ALS as a multisystem brain degeneration disorder instead of a disease limited to motor neurons. Advanced neurophysiological studies can help in identifying the sensory-motor impairment in many different diseases. Transcranial magnetic stimulation (TMS) is a non-invasive way to stimulate nerve cells in the superficial brain areas by applying a high-energy magnetic field at the skull surface, which produces a perpendicular-induced electrical field through the cortex.

Recently, a surge in interest has been recorded in electrophysiological techniques that induce short-term changes in the human cortex excitability, such as patterned electrical or mechanical muscle and nerve stimulation, and in methods that indirectly stimulate brain regions through transient magnetic fields or weak electrical currents (19–21). Within this context, paired associative stimulation (PAS) has drawn attention both as a therapeutic intervention (22, 23) and as an experimental method to investigate Hebbian principles of synaptic plasticity. In the prototypical form of the PAS (24), an electrical stimulus is administered to a peripheral nerve in advance of a magnetic stimulus delivered to the contralateral primary motor cortex (M1). The interstimulus interval is adjusted to ensure that inputs to M1 from the afferent volley arising from the nerve stimulation occur simultaneously with the magnetic stimulation. Repeated pairing of the two sources of stimulation (i.e., association) over an extended time increases the excitability of corticospinal projections from M1. A reduction in corticospinal excitability has been reported when the interstimulus interval is adjusted to allow a corollary of the afferent volley to reach M1 after the magnetic stimulus (25).

This neuroplastic adaptation revealed by the PAS appears to exhibit several criteria designated for long-term potentiation (LTP) and long-term depression (LTD): its effects evolve quickly, are reversible, and persist beyond the period of stimulation (26–28). Pharmacological agents interacting with N-methyl-D-aspartate (NMDA)-receptor activity interfere with the outcomes of the PAS, thus supporting the hypothesis that LTP-like changes are implicated (28).

Since the first description of this technique by Stefan et al. (24), there has been a wide range of derivative investigations concerning, among other features, the most effective interstimulus intervals (ISIs) (29, 30), the muscles in which the effects can be elicited (24, 31, 32) and the extent to which they can be induced in various clinical populations (23). Moreover, large interindividual differences in response to the PAS have been observed (33). This led to investigations on potential mediating factors, such as age (34), cortical anatomy (35), and the role of specific genetic polymorphisms (36, 37). The PAS protocol was tested in patients with ALS only in two trials; one of them (38) recently demonstrated that sensory-motor networks are also hyperexcited and Riluzole, one of the approved drugs for ALS therapy, can positively modulate this aspect. Physical training could positively or negatively modulate this sensory-motor hyperexcitability in ALS. Several studies on the animal model report neuroprotective effects induced by moderate physical exercise (running), mainly ascribed to a protective role on astrocytes (39); on the contrary, other studies do not show differences between the survival of mice that make exercise with different intensities (40). Finally, other studies report a worsening of the clinical course in the mouse model practicing intense physical exercise (41).

Studies on a mouse model, which practiced physical exercise in water (swimming), report an improvement in the clinical course characterized by reduction of symptoms and increased survival (42, 43). This effect has been hypothesized to be related to the repetitive activation of the same neuronal circuits and the subsequent action on the transcriptome of the activated neurons, which would induce a neuroprotective effect.

Many other articles recommended swimming training in ALS (44–46). The difference in course and survival found in the swimming mouse compared to the running one makes us wonder about the reasons for such different clinical courses and what are the peculiar characteristics of physical activity in water able to mediate neuroprotection.

Among the mechanisms, it could be hypothesized that different frequencies of the firing rate of the motor neuron could lead to neuroprotective or neurodegenerative phenomena; moreover, in the water exercise, the body undergoes a continuous skin stimulation, which can modulate cortical excitability, as experienced in persons with spinal cord injury (47). The effects of sensory stimulation have been widely described for whole-body vibration (WBV). WBV is a mechanical stimulation technique of the body mainly used for study purposes to evaluate the induced neuromuscular responses; it is also used in the rehabilitation of patients with stroke or spinal injuries (47–49). The effects of vibration on muscle strength, motor coordination, and postural control are widely documented, consisting of a temporary sustained enhancement of corticospinal excitability concomitant with spinal inhibition (47). However, the physiological basis of these effects is still unknown. Mechanisms modulating neuronal excitability at the spinal and supraspinal levels have been supposed, but the results obtained from the different studies are still discordant. Different results are probably due to vibratory frequencies used (49).

The vibration also determines a facilitatory effect on the motor evoked potential (MEP) of the stimulated territory (48, 50) and a decrease of Hoffmann's reflex (H-reflex) (49, 51–56), which represents the electrophysiological equivalent of the myotatic reflex. The ability of the vibration to modulate the H-reflex could act at the spinal level, but a cortical involvement cannot be excluded (48, 49).

The increase in the amplitude of the MEP following WBV, documented in several articles, represents an increase in the excitability of the corticospinal projections and, therefore, an action at a supraspinal level (48). Nevertheless, Sayenko et al. (55) demonstrated that also patients with spinal cord injury had a reduction of H-reflex after WBV, suggesting a direct action of vibration on the lower motor neuron excitability.

The effects of the PAS on the cortical excitability, as observed above, are synapse specific and are restricted to cortical representations of the muscles innervated by the peripheral nerve that has been electrically stimulated (57). Thus, vibration, which can activate different sensory regions, does not appear to be the best sensory cue to study the sensory-motor networks evaluated by the PAS.

This trial aimed to demonstrate the effects of tactile stimulation on the sensory-motor network's excitability in healthy subjects, to determine whether it should be encouraged or not in the rehabilitation of patients with ALS.

## Materials and Methods

### Skin Stimulation and Its Effects on the Paired Associative Stimulation

In total, 15 healthy volunteers were recruited. The exclusion criteria were as follows: drowsiness (assessed with the Epworth sleepiness scale), alcohol and coffee consumption, or the intake of drugs capable of interacting with the neuronal excitability threshold in the last 24 h, women in the menstrual or premenstrual phase (58), high resting motor threshold (RMT) (exceeding the maximum TMS output power to be elicited), pregnant women, subjects with epilepsy, and subjects with metal implants (e.g., pacemakers and hearing aids).

Each participant in this study underwent two sessions: in the first session, he underwent the standard PAS protocol, while in the second session, he underwent the PAS protocol after 10 min of skin stimulation (brushing).

### Standard Paired Associative Stimulation Session

**Figure 1:**
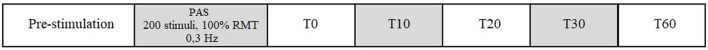
The standard paired associative stimulation (PAS) session protocol.

The following parameters were recorded for each subject (Figure 1):

- Resting motor threshold (RMT), belly-tendon registration from the abductor pollicis brevis (APB) of the non-dominant hand.
- Maximal amplitude of the motor evoked potential (MEP) from the APB of the non-dominant hand; TMS output was gradually increased by 10% until the maximal peak-to-peak amplitude was recorded from the APB.
- Sensory electrical threshold of the median nerve of the non-dominant hand. The sensory threshold was defined as the minimal intensity of stimulation that can be perceived (59) in 5 out of 10 tests.
- Latency of the N20 component of the somatosensory evoked potential (SSEP) from the stimulation of the median non-dominant nerve (5 trials of 200 stimuli, filters set as 5/3,000 Hz, stimulus rate 3.3 Hz, stimulation with monophasic rectangular pulses with a duration of 0.1 ms, intensity 1.3 times the motor threshold, recording electrode between the central and contralateral parietal electrode, and referenced to the ear electrode) (60).
- Average MEP amplitude from the APB of the non-dominant hand obtained through the delivery of 10 stimuli at 0.1 Hz with an intensity of 120% of the RMT. This average amplitude was recorded before the PAS stimulation, immediately after (T0) and after 10 (T10), 20 (T20), 30 (T30), and 60 (T60) min from the PAS stimulation.
- Amplitude and latency of the median compound muscle action potential (cMAP) of the non-dominant hand.

For the assessment of the RMT, the minimum intensity of transcranial magnetic stimulation able to evoke a motor evoked potential with an amplitude at least of 50 μV in at least 5 out of 10 stimulations recorded at the level of the APB of the non-dominant hand was considered. The non-dominant hand was tested to avoid possible entrapment syndrome, more frequent in the dominant hand.

The TMS was performed through a high-frequency biphasic magnetic stimulator (Magstim Rapid—The Magstim Company Ltd., Whitland, Southwest Wales, United Kingdom) connected to an eight-shaped coil. The particular shape of the coil allows the delivery of a very focused stimulation (61).

The PAS stimulation was performed by administering 200 pairs of stimuli at a frequency of 0.3 Hz, with the same protocol previously used (38). The magnetic stimulus was delivered *via* TMS in the hotspot for the APB of the contralateral hand, at an intensity of 100% of the RMT; the eight-shaped coil was placed tangentially to the scalp with an angle of approximately 45° to the midline. The hotspot was considered the location that evokes the largest electromyogram (EMG) responses while applying a series of pulses at a relatively high intensity (62). The magnetic stimulus was coupled to an electrical stimulus delivered on the median nerve of the non-dominant hand (contralateral to the magnetically stimulated cortex); the electrical stimulation was administered at 300% of the previously recorded sensory electrical threshold, with a duration of 500 μs. However, the duration of the electrical stimulus was adjusted to maintain the stimulus always below the motor threshold, to avoid any activation of muscle fibers of the APB, which would provide further activation of an afferent proprioceptive volley, thus possibly modifying the effect of the PAS.

The interstimulus interval (ISI) between electrical and magnetic stimuli was calculated by adding 6 ms to the N20 latency of the somatosensory evoked potentials (SSEPs) of the stimulated hand so that the inputs afferent to the primary motor cortex deriving from the electrical stimulation of the median nerve precede the cortical stimulation obtained with TMS (63).

### Paired Associative Stimulation Protocol With Skin Stimulation

**Figure 2:**
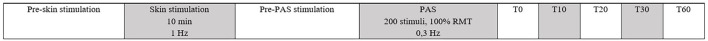
The PAS protocol with skin stimulation.

In the second session, tactile skin stimulation was performed before the PAS stimulation (Figure 2).

A skin mechanical stimulation rather than an electrical stimulation was carried out to activate only the sensitive Aβ and not small sensitive Aδ or C fibers. Unlike other studies, we used a mechanical tactile stimulation instead of a vibratory stimulation because the latter let the entire hand vibrate, thus stimulating other nervous areas such as the ulnar or radial ones. We speculate that the effects of the PAS can be modulated by sensory stimuli delivered to the same sensory area.

Skin stimulation was performed by brushing through the bristles of a brush, with a stimulation frequency of 1 Hz for 10 min in the territory innervated by the median nerve of the non-dominant hand.

The following parameters were recorded both before and after skin stimulation:

- Motor threshold at rest, belly-tendon registration from the APB of the non-dominant hand.
- Maximal amplitude of the motor evoked potential from the APB of the non-dominant hand; TMS output was increased with steps of 10% of stimulation output until the maximal peak-to-peak amplitude was recorded from the APB.
- Sensory electrical threshold of the median nerve on the non-dominant hand.
- Average amplitude of the MEP after 10 stimuli of TMS at 120% of the RMT and a frequency of 0.1 Hz (registration from the APB of the non-dominant hand).

Subsequently, each subject was subjected to the PAS stimulation with 200 pairs of stimuli delivered with a frequency of 0.3 Hz (same protocol as the standard PAS session). Average MEP amplitude from the abductor pollicis brevis (APB) of the non-dominant hand was obtained through the delivery of 10 stimuli at 0.1 Hz, with an intensity of 120% of the RMT. This average amplitude was recorded immediately after the PAS stimulation (T0) and after 10 (T10), 20 (T20), 30 (T30), and 60 (T60) min.

### F-Wave Amplitude Study

On a different day from the two PAS sessions, the same skin stimulation was performed as in the PAS protocol with skin stimulation, and the average amplitude of the F-wave was obtained by 10 supramaximal stimuli of the non-dominant median nerve at 1 Hz before the 10-min skin stimulation, immediately after (T0) and after 10 (T10), 20 (T20), 30 (T30), and 60 (T60) min.

This study complied with the Declaration of Helsinki and international safety guidelines and was approved by the local ethics committee. All the subjects provided written informed consent for their participation in this study.

## Statistics

Parametric tests were used when Levene's test demonstrated equal variances of the sample. In particular, the comparison between RMT and the mean baseline MEP before the standard PAS session and before and after skin stimulation in the PAS protocol with skin stimulation was performed with the Student's *t*-test. The MEP amplitude and F-wave amplitude in the different time points were compared through the repeated measures-ANOVA (RM-ANOVA), with a within-subject analysis comparing the single parameter in the different time points and a between-subject analysis comparing the parameters in the two PAS sessions.

Statistical significance was defined with a *p*-value < 0.05. All the statistics were performed with IBM SPSS statistics version 25.

## Results

In total, 15 volunteers were enrolled, 9 women (60%) and 6 men (40%), aged between 20 and 62 years (mean age: 28.87 ± 10.67 years).

The mean RMT recorded in the first trial (the standard PAS session) was 56.67 ± 7.08%; the mean RMT in the PAS protocol with skin stimulation was 56.13 ± 7.55% in the preskin stimulation session and 57.40 ± 8.47% in the postskin stimulation session.

For the electric sensory threshold, an average value of 1.87 ± 0.52 mA was calculated in the first trial and an average value of 1.93 ± 0.80 mA was calculated in the second trial.

The mean MEP amplitude calculated at the different time points during the first and second trials is shown in Table 1.

**Table 1:** Mean RMT, sensory threshold, and the MEP amplitude ± SD in the different time points, both with and without skin stimulation trials.

	**Standard PAS**	**PAS protocol with**	
	**session**	**skin stimulation**	
		**Pre-skin**	**Post skin**
	**stimulation**	**stimulation**	
RMT (%)	56,67 ± 7,08	56,13 ± 7,55	57,40 ± 8,47
Sensory threshold (mA)	1,87 ± 0,52		1,93 ± 0,80
MEP pre-PAS (mV)	0,69 ± 0,62	0,59 ± 0,27	0,53 ± 0,30
MEP T0 (mV)	0,57 ± 0,56		0,52 ± 0,23
MEP T10 (mV)	0,52 ± 0,34		0,58 ± 0,28
MEP T20 (mV)	0,77 ± 0,75		0,77 ± 0,43
MEP T30 (mV)	0,69 ± 0,42		0,91 ± 0,66
MEP T60 (mV)	0,59 ± 0,74		0,74 ± 0,82

*RMT, resting motor threshold; MEP, motor evoked potential; PAS, paired associative stimulation*.

No statistically significant changes (*p* > 0.05) were demonstrated in the RMT recorded in the three detections (baseline RMT in the standard PAS session trial and before and after skin stimulation in the PAS protocol with skin stimulation).

The mean baseline MEPs were also compared between the three detections and no significant differences were found (*p* > 0.05).

The presence of significant global changes in the mean MEP amplitude in the different time points (baseline, T0, T10, T20, T30, and T60) was assessed by RM-ANOVA, both in the standard PAS session and PAS protocol with skin stimulation. No significant changes in the global mean MEP amplitude either in the first trial [F _(2.783, 38.967)_ = 1.012, *p* > 0.05] or in the second trial [F _(1.828, 18.282)_ = 2.016, *p* > 0.05] were found.

The within-subject analysis in the standard PAS session highlighted a significant increase in the amplitude of the MEP after the PAS at T20 (*p* = 0.045).

The within-subject analysis in the PAS protocol with skin stimulation showed a statistically significant increase in the mean MEP amplitude at T30 (*p* = 0.027) and T60 (*p* = 0.031) compared to the baseline MEP recorded before the PAS and after skin stimulation. In the other time points, no significant differences were found.

**Figure 3:**
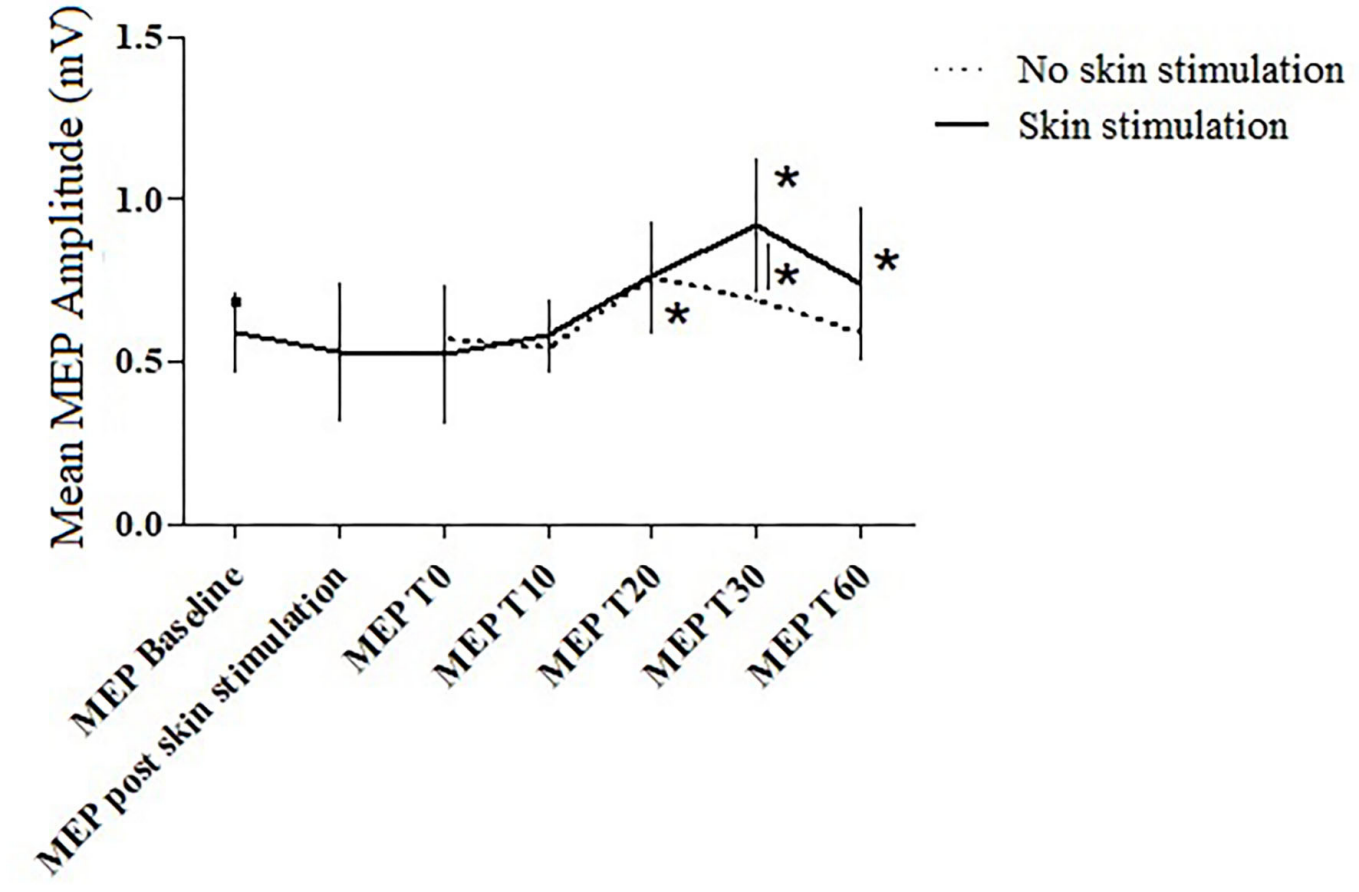
The MEP amplitude in the two trials at the different time points. Error bars indicate the SEM. *= *p* < 0.05.

**Figure 4:**
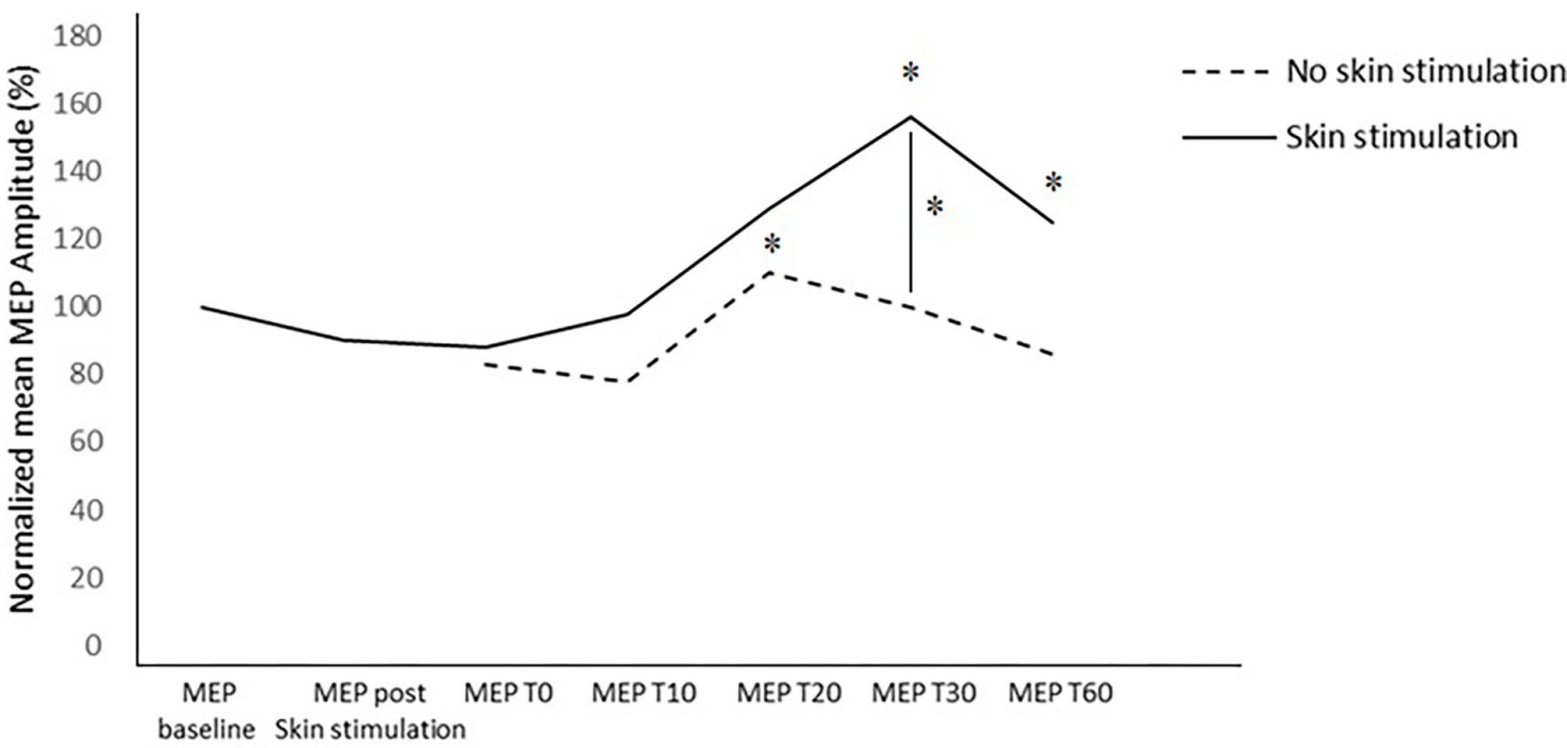
Normalized MEP amplitude in the two trials at the different time points. *= *p* < 0.05.

In the between-subject analysis, significance was achieved for a higher increase in the MEP amplitude at T30 (*p* = 0.031) in the PAS protocol with skin stimulation compared to the standard PAS session; a trend to significance was also found at T60 (*p* = 0.072) (Figures 3, 4).

Repeated measures-ANOVA showed no differences between F-wave amplitude before and after skin stimulation at the different time points (*p* > 0.05; Table 2).

**Table 2:** Average F-wave amplitude ± SD in the different time points.

	**Average F wave amplitude**
pre-skin stimulation	0,17 ± 0,10
T0	0,12 ± 0,07
T10	0,13 ± 0,05
T20	0,14 ± 0,06
T30	0,13 ± 0,06
T60	0,15 ± 0,07

## Discussion

Skin stimulation was investigated in a healthy subject sample and the effects on transcortical excitability were evaluated through the PAS protocol. The effects of skin stimulation were tested on the healthy subjects to infer the effects of sensory cues during rehabilitation on the transcortical sensory-motor excitability. Previous studies (38) demonstrated an increased transcortical sensory-motor excitability in patients with ALS after the PAS protocol, thus suggesting possible NMDA-mediated excitotoxicity not only in the primary motor cortex but also in the transcortical sensory-motor networks. Moreover, the effects of Riluzole, the main approved drug for ALS, do not act only on the primary motor cortex excitability (64–66), but also on the sensory-motor projections.

Many factors can modify cortical excitability, increasing or decreasing it and leading to a beneficial or deleterious effect in patients with ALS. Studies on G93A-SOD1 mice, the experimental animal model for ALS, showed a significant difference in course and prognosis between in-water and extrawater exercise, with the former associated with increased survival (42, 43). Among the possible hypothesized mechanisms, the tactile stimulus associated with swimming was evaluated in this trial. While swimming, in fact, the continuous resistance opposed by water determines a constant skin stimulation on the body surface.

Sensory stimulation is already recognized as an important tool for rehabilitation in many neurological diseases such as stroke and spinal cord injuries (47, 67), diseases characterized by downregulation of the pyramidal tract. Several studies (47–49) investigating the neurophysiological effects of sensory stimulation, usually vibration, report a reduction of the lower motor neuron excitability.

However, supraspinal effects of vibration have also been reported (48), with an increased amplitude of the MEP, suggesting an upmodulated cortical excitability.

This trial aimed to evaluate the primary motor cortex and sensory-motor networks' excitability after skin stimulation, to confirm or deny a role for sensory stimulation during rehabilitation in diseases characterized by transcortical hyperexcitability, such as ALS.

At the beginning of each of the two stimulation sessions, with and without skin stimulation, the baseline RMT and MEP amplitude were evaluated to check any change in intracortical excitability between the first and the second sessions. Indeed, no significant changes were found for these parameters.

The absence of differences in RMT values before and after skin stimulation shows that the latter does not induce changes in the NMDA-mediated excitability of the primary motor cortex (68).

In the standard PAS session, the MEP amplitude increased at T20 (*p* = 0.045), compared to the baseline value; in the PAS protocol with skin stimulation, a significant increase in the MEP amplitude was found at T30 (*p* = 0.027) and T60 (*p* = 0.031) compared to the baseline.

No changes in F-wave amplitude were found at any time point after skin stimulation compared to the baseline.

Furthermore, the between-subject analysis showed a significantly higher increase in the mean MEP amplitude in the PAS protocol with skin stimulation compared to the standard PAS session at T30 (*p* =0.031) and a trend toward significance at T60 (*p* = 0.072).

The skin stimulation was able to further increase sensory-motor networks' susceptibility to the PAS and extend the duration of the effect (at least 60 min). In this way, we can confirm an effect on a supraspinal level for sensory stimulation, acting not just on the primary motor cortex but also on the sensory-motor networks.

The F-wave amplitude did not change before and after the skin stimulation at any time point, not even in the same ones (T30), which demonstrated an increased MEP after the standard (T30) and the PAS protocol with skin stimulation. The F-wave amplitude is related to the intrinsic primary motor cortex excitability, with increased excitability expected to reduce the amplitude. This is to confirm that the skin stimulation does not directly modify the primary motor cortex excitability, but increases the excitability of the sensory-motor networks. Moreover, we can speculate that the F-wave represents the tonic and intrinsic excitability of the primary motor cortex, while the MEP amplitude preferentially represents the excitability of the interneurons rather than motor neurons (69, 70).

When compared to the evidence from previous articles (38), a similar behavior between skin-stimulated healthy controls and patients with ALS can be highlighted, with a higher and longer-lasting increase in the MEP amplitude after the PAS.

The results of this study should discourage the use of sensory cues in the rehabilitative protocols for patients with ALS, given the possibility of further increasing the transcortical excitability, thus potentially increasing the excitotoxic damage. Moreover, the synergistic effect on cortical excitability of the PAS coupled with skin stimulations confirms a peculiar role for spatial summation in the medium-term brain modulation and associative learning.

On the other hand, the evidence of a better prognosis for mice making in-water physical exercise (42, 43) cannot be explained by the continuous body skin stimulation by water. Then, the intensity of physical exercise during the swimming activity can be taken into account. Other authors demonstrated a better prognosis for ALS when a moderate exercise was performed (71–73); in particular, Carreras (73) demonstrated a reduced motor neuron loss in ALS mice when moderate exercise was performed, compared to sedentary and high-level exercise. The authors concluded that different intensities of exercise could have different outcomes in ALS mice prognosis; moderate level exercise, compared to a sedentary lifestyle, could increase the release of neurotrophic factors, such as brain-derived neurotrophic factor (BDNF), insulin-like growth factor-1 (IGF-1), and vascular endothelial growth factor (VEGF), with a positive role on motor neurons survival. High-level exercise may be a combination of the beneficial effects of exercise together with the negative effects of stressful excursion, with an increase in glutamate and reactive oxygen species release.

The main limitation of this study was the absence of a study arm with patients with ALS. In this study, patients with ALS would have been involved in further trials, if the sensory stimulation had induced a negative modulation of the sensory-motor networks. In fact, this study group already demonstrated hyperexcitability of these transcortical projections in ALS (38). The results of this trial are inhibiting further investigations on patients for ethical reasons.

If the skin tactile stimulation should be avoided in ALS patients' physical activity, other diseases and conditions could benefit from it. Skin stimulation should be encouraged in pathologies characterized by reduced cortical excitability, such as stroke (74) or remitting phase of multiple sclerosis (75). In these diseases, some evidence for sensory stimulation during physical activity is already available, regardless of the sensory mode used (76–78) and even for dysphagia (79–81). The administration of sensory cues during the training already provided evidence for benefit, also in the non-motor rehabilitation; protocols using vestibular, somatosensory, and optokinetic stimulation each have been shown to produce transient improvements in visuospatial neglect (82).

## Conclusion

We can conclude that sensory cues during rehabilitation can be encouraged in diseases characterized by cortical and transcortical hypoexcitability, but should be avoided in patients with ALS.

## Data Availability Statement

The original contributions presented in the study are included in the article/supplementary material, further inquiries can be directed to the corresponding author.

## Ethics Statement

The studies involving human participants were reviewed and approved by Policlinico Umberto I. The patients/participants provided their written informed consent to participate in this study.

## Author Contributions

MC: design and drafting of the study. CC and LL: interpretation of data for the study and revising it critically for important intellectual content. GT: acquisition of data for the study and revising it critically for important intellectual content. FM: analysis of data for the study and revising it critically for important intellectual content. MG: acquisition, analysis, or interpretation of data for the study, and revising it critically for important intellectual content. MI: conception of the study and revising it critically for important intellectual content. All authors contributed to the article and approved the submitted version.

## Conflict of Interest

The authors declare that the research was conducted in the absence of any commercial or financial relationships that could be construed as a potential conflict of interest.

## Publisher's Note

All claims expressed in this article are solely those of the authors and do not necessarily represent those of their affiliated organizations, or those of the publisher, the editors and the reviewers. Any product that may be evaluated in this article, or claim that may be made by its manufacturer, is not guaranteed or endorsed by the publisher.
